# Mechanisms of genotoxin-induced transcription and hypermutation in *p53*

**DOI:** 10.1186/1475-2867-6-27

**Published:** 2006-12-01

**Authors:** Barbara Wright, Jacqueline Reimers, Karen Schmidt, Evan Burkala, Nick Davis, Ping Wei

**Affiliations:** 1 Division of Biological Sciences, The University of Montana, Missoula, MT 59812, USA

## Abstract

It is widely assumed that genotoxin-induced damage (e.g., G-to-T transversions) to the tumor suppressor gene, *p53*, is a direct cause of cancer. However, genotoxins also induce the stress response, which upregulates *p53 *transcription and the formation of secondary structures from ssDNA. Since unpaired bases are thermodynamically unstable and intrinsically mutable, increased transcription could be the cause of hypermutation, and thus cancer. Support for this hypothesis has been obtained by analyzing 6662 mutations in all types of cancer compared to lung and colon cancers, using the *p53 *mutation database. The data suggest that genotoxins have two independent effects: first, they induce *p53 *transcription, which increases the number of mutable bases that determine the incidence of cancer. Second, genotoxins may alter the *fate*, or ultimate mutation of a mutable base, for example, by causing more of the available mutable Gs to mutate to T, leaving fewer to mutate to A. Such effects on the fate of mutable bases have no impact on the incidence of cancer, as both types of mutations lead to cancer.

## Background

### Base damage to *p53*

The wild-type p53 protein inhibits neoplastic transformations as well as tumor growth. Moreover, if DNA damage occurs, p53 is induced and maintains the integrity of the genome by causing cell cycle arrest, to allow for repairs or, in the event of severe damage, by sacrificing the damaged cells (apoptosis). More than half of all cancers are associated with mutations in the *p53 *gene [1-3], and the vast majority of these occur in the sequence-specific DNA binding domain. DNA strand breakage and genetic instability are generally thought to arise as a result of direct base damage by genotoxic stressors, such as reactive oxygen species, which are not only in the environment but are formed *in vivo *under normal physiological conditions. An extensive literature documents the effect of genotoxins directly on the *p53 *gene [4,5]. Of particular importance in the present context are genotoxin-induced G:C-to-T:A transversions that occur at high frequencies in some cancers [6-8]. While these transversions are relatively rare in most mutable genes, they represent a "signature" *p53 *mutation in experiments which document DNA damage by carcinogens. These studies appear to implicate base damage as the direct cause of *p53 *mutations, and to suggest that such damage increases the incidence of lung cancer.

However, severe base damage results in apoptosis *in vivo*, and is unlikely to foster the creation of robust cancer cells. A more conducive environment for the development of cancer may emerge from a response to relatively mild DNA damage that activates stress response mechanisms [9] and upregulates *p53 *transcription. Transcription drives secondary structures which expose unpaired bases for hypermutation at specific sites ("hot spots"). Mutations in *p53 *inactivate the protein, which regulates interactions including the coordination of DNA repair with the rate of cell growth. These relationships will be specifically disrupted without significant damage to metabolism in general, providing ideal conditions for the creation and selection of cells with high rates of cell division, thus leading to tumorigenesis. This "directed" mutagenic mechanism, dependent upon the stress response rather than random base damage, is more likely to be responsible for the evolution of hypermutable bases in critical regulatory genes such as *p53*.

### The stress response

A number of investigations in the microbial world have provided evidence that environmental stressors derepress and/or activate transcription specifically in genes that must mutate to overcome the stress [10,11]. The implications of this mutagenic mechanism for evolution are profound [12,13]. The increased rate of stress-induced transcription based on mRNA accumulation and half-life has been correlated with mutation rates, and predicted effects of promoter strength and supercoiling on mutation frequencies have been demonstrated in prokaryotes [10,14,15]. In higher organisms, only a few studies supporting this mechanism are available [16,17]; also, convincing evidence exists for a direct dependence of mutation frequency on the rate of transcription during somatic hypermutation in the immune response [18].

Genotoxic stressors cause strong promoter activation and upregulation of *p53 *transcription, which could increase *p53 *mutation frequencies. The persistent over-expression of p53 protein is commonly associated with the accumulation of *p53 *mutations and tumorigenesis [19,20]. The cellular stress response involves a large number of genes in a complex network, and genotoxins induce transcriptional upregulation of *p53 *by both indirect and direct means. DNA-damaging genotoxins elicit a strong p53 protein-dependent response resulting in upregulation of *p53 *transcription [9]. This activation of transcription occurs either by direct interaction with the basal *p53 *promoter [21] or by indirect upregulation, which typically involves activation by the ubiquitous transcription factor, NF-κB. Specifically, benzo [*a*]pyrene, a potent genotoxic component of cigarette smoke, upregulates *p53 *transcription via induction of NF-κB [22]. As the *p53 *gene is responsive to many stress-related signals [23,24], its expression is upregulated by a variety of genotoxic stressors. The activation of *p53 *transcription will drive supercoiling, which creates and stabilizes ssDNA secondary structures containing unpaired bases that are intrinsically mutable [25,26]. Therefore transcription, *per se*, could promote hypermutation and ultimately, cancer.

### Intrinsically mutable bases

Unpaired bases of ssDNA are thermodynamically unstable, and point mutations occur by known chemical mechanisms having finite, significant activation energies under physiological conditions. For example, the hydrolytic deamination of cytosine in ssDNA occurs 160 times more frequently than in dsDNA, and CpG sequences are methylated non-enzymatically by S-adenosylmethionine, rendering them 40 times more susceptible to deamination than non-methylated sequences [26]. In the *p53 *gene, most of the hypermutable bases are methylated CpGs. Two major non-enzymatic mutagenic events, the hydrolytic deamination of cytosine and the oxidation of guanine, are estimated to occur 100–500 times per day in each human cell, and 2000–10000 purine bases turn over per day in each cell due to hydrolytic depurination and repair. Thus, G and C are much more mutable by these mechanisms than A and T, and, because of its size, A is more likely than C or T to replace G at apurinic sites [26,27]. In human genetic disease, 35% of single base mutations are in CpG dinucleotides and over 90% are C-to-T or G-to-A mutations occurring at a frequency 42-fold higher than that predicted by chance [28]. Thus, these are the primary "default" or background mutations in ssDNA that are determined by the inherent chemical instability of each base and the extent to which it is exposed during events such as replication and transcription.

Transcription-induced intrinsic mutagenesis, due only to the extent to which a base is unpaired in ssDNA, correlates with mutation frequencies in *p53 *[17]. Interestingly, this circumstance would be difficult to distinguish from one that is also dependent upon, and therefore superimposed upon base exposure; for example, genotoxin-induced G-to-T transversions, and enzyme-catalyzed mutations, both of which are more effective on ssDNA than dsDNA.

### Predicting base mutability *in vivo*

In previous studies a computer algorithm (*mfg*) has been used to simulate the formation of secondary structures in ssDNA during transcription and predict the relative mutability of unpaired bases in these structures [11]. This program performs a sliding window analysis of any given sequence, in which a chosen length of nucleotides is folded successively, beginning with each base in the sequence. *Mfg *interfaces with another computer program (*mfold*) that reports all possible secondary structures that can form from each folded segment, in order of their stability. The *mfg *program predicts (i.e., calculates) the mutability of each unpaired base from two key variables: a) the stability of the most stable secondary structure in which the base is unpaired, and b) the percent of total folds in which it is unpaired. The Mutability Index (MI) is the product of these two factors: the stability (-ΔG) of the most stable SLS multiplied by percent unpaired.

**Figure 1:**
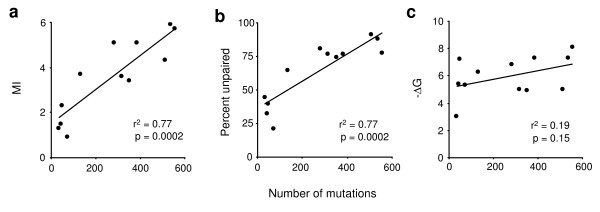
Linear regression analyses of the correlations between predicted and observed mutation frequency in *p53*. The mutations analyzed are the first two positions of codons 175, 245, 248, 249, 273, and 282. Mutation frequencies are correlated with: (**a**) MI, the base mutability index; (**b**) Percent unpaired, the fraction of total folds during transcription in which each mutable base is unpaired; and (**c**) -ΔG, the stability (kcal/mol) of the most stable secondary structure in which each base is unpaired. Data on the y-axis are obtained from the *mfg *computer program that simulates transcription [17].

The computer algorithm, *mfg*, has been used to analyze hypermutable codons in *p53 *[17]. Using the non-transcribed strand, twelve hypermutable bases (the first two positions of codons 175, 245, 248, 249, 273, and 282) were analyzed (a total of 3492 mutations), and the computer-predicted MIs were found to correlate (r^2 ^= 0.77; p = 0.0002) with the mutation frequencies. No correlation was found using the transcribed strand (r^2 ^= 0.27; p = 0.1). To determine the relative contribution of percent unpaired and -ΔG to MI, all three are plotted independently against mutation frequency (Fig. 1). Secondary structure stability shows no correlation with mutation frequency (Fig. 1c) while the correlation with percent unpaired (Fig. 1b) is comparable to that of MI (Fig. 1a). Therefore, the extent to which a base is unpaired during transcription is a much better predictor of base mutability (in *p53*) than the stability of the most stable structure in which it is unpaired. Another variable that can be analyzed by this program is the effect of transcription level, which correlates with the amount of RNA transcript and ssDNA formed [29,30]. Over a broad range of transcription levels (> 40 nt window size) predicted mutation frequencies increase incrementally, suggesting a threshold mechanism for inducing the level of transcription in *p53 *that leads to mutagenesis.

As the implications of the previous analyses are not yet widely recognized, an original analysis of the *p53 *mutation database is presented here. These data provide strong support regarding the roles of genotoxins, base damage, and transcription with respect to the incidence of cancer. Thus, new lines of evidence support the conclusion that genotoxins: (1) activate *p53 *transcription and hypermutation, which increases the number of mutable bases and thus the incidence of cancer, and (2) alter the *fate *of mutable bases, which does not affect the incidence of cancer.

## Results and discussion

A systems analysis of 6662 mutations in the International Agency for Research on Cancer TP53 Mutation Database (version R4) [31,32] has revealed relationships and mechanisms not easily examined in the laboratory. Due to the rarity of mutations, a large database is essential for obtaining reliable values in assessing the origin and fate of mutable bases in cancers. Moreover, these mutations occurred under physiological conditions, whereas experimental conditions for analyzing effects of genotoxins *in vitro *may not reflect conditions *in vivo*. In our analyses, the dual role of genotoxins was discovered only in light of two kinds of information extracted from the same large *p53 *mutation database: first, genotoxins induce a stress response that upregulates *p53 *transcription and mutation frequency to a comparable extent in lung cancer as in all types of cancer; this frequency determines the incidence of cancer. Second, base damage by genotoxins increase the G-to-T versus the G-to-A ratio in lung cancer; however, this has no effect on the incidence of cancer. This key distinction is essential to understanding the mechanisms involved.

Table 1 summarizes the relative number of mutations in the four bases and Tables 2 and 3 show the fates of G and C mutations in codons 210–290 of *p53 *for all types of cancers (6662 total mutations, including lung), compared to lung cancer mutations exclusively (875). The relative percent of mutations in the four bases is remarkably similar in lung and in all cancers, especially in view of the presumed effect of genotoxins on mutations in lung tissue (Table 1). The most striking observation is that, although the percent of G mutations in lung cancers is comparable to the percent in all types of cancers (55.3% versus 50.7%), the base to which G most frequently mutates is clearly different in the two data sets (Table 2). Of the total G mutations in all cancers (including lung), 61.7% are to A and 27.9% are to T, whereas in lung cancer mutations, 31.6% of G mutations are to A and 53.1% are to T. Thus, the ultimate fate of mutable G bases in lung cancers appears to be strongly influenced by the unusually potent genotoxic agents in smoke. The observation that G-to-A default mutations decrease to approximately the same extent as G-to-T mutations increase suggests that these mutations share ("compete" for) the same source of available background mutable Gs. If lung cancers were excluded from "all cancers", it would not be possible to depict the competition in all cancers between (default-induced) G-to-A mutations and (genotoxin-induced) G-to-T mutations for the (limited number of transcription-induced) available mutable Gs. The relative percent of mutations in the four bases is similar in lung and in all mutations, suggesting a common regulatory mechanism that is different from that which determines the fate of G mutations. Note that a distinction is made between base mutability, or the *propensity *of a base to mutate (determined by transcription), and the *ultimate fate*, or final mutation that occurs (dependent upon the absence or presence of a mutagen). It is the number of mutable bases that correlate with the incidence of cancer. Having distinguished between the propensity of a base to mutate and the fate or final outcome of a mutation, it should be noted that lung mutations are characterized by a minor increase in the number of mutable Gs (4.6%) and As (1.4%), which could be critical to the incidence of this type of cancer.

**Table 1:** Mutations in lung compared to all cancers (including lung) in *p53 *codons 210–290

	A	C	**G**	T
Total number of all mutations in 240 nt (6662)	865	1875	**3375**	547
Percent of total	13.0%	28.1%	**50.7%**	8.2%
				
Number of lung mutations in 240 nt (875)	126	198	**484**	67
Percent of lung	14.4%	22.6%	**55.3%**	7.7%
				
Difference in total mutations compared to lung mutations	+1.4%	-5.5%	**+4.6%**	-0.5%

All point mutations in codons 210–290 were analyzed.

**Table 1 **shows the total number of all mutations (including lung) compared to lung mutations and the propensity of each of the four bases to mutate to any other base. This sequence is composed of 56 As, 66 Cs, 67 Gs, and 54 Ts. Mutations of G are shown in bold.

**Table 2:** Mutations of G to other bases in *p53 *codons 210–290

	**A**	C	**T**
Total G mutations including lung (3375)	**2082**	351	**942**
Percent of total	**61.7%**	10.4%	**27.9%**
			
Lung G mutations (484)	**153**	74	**257**
Percent of lung	**31.6%**	15.3%	**53.1%**
			
**Difference in total G mutations due to the presence of lung G mutations**	**-30.1%**	+4.9%	**+25.2%**

All point mutations in codons 210–290 were analyzed.

**Table 2 **shows the ultimate fate of G mutations in all cancers due to lung cancers, with G-to-A and G-to-T mutations in bold.

**Table 3:** Mutations of C to other bases in *p53 *codons 210–290

	A	G	T
Total C mutations including lung (1875)	157	187	1531
Percent of total	8.4%	10.0%	81.6%
			
Lung C mutations (198)	21	31	146
Percent of lung	10.6%	15.7%	73.7%
			
Difference in total C mutations due to the presence of lung C mutations	+2.2%	+5.7%	-7.9%

All point mutations in codons 210–290 were analyzed.

In **Table 3**, the ultimate fate of C mutations in all cancers due to the presence of lung cancers is shown.

**Figure 2:**
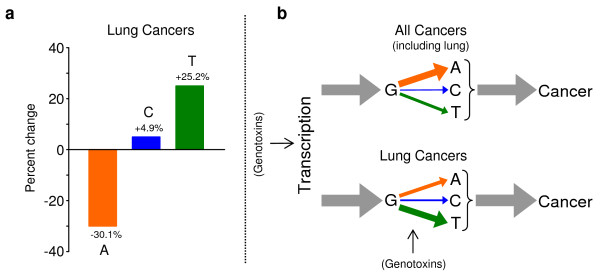
Visual depictions of the frequencies and fates of G mutations due to lung cancers in *p53*. (**a**) In lung cancer mutations compared to all cancers, there is a decrease of 30.1% (orange) in G-to-A mutations, an increase of 25.2% (green) in G-to-T mutations, and an increase of 4.9% (blue) in G-to-C mutations. (**b**) The relative number or frequency of G mutations (gray), resulting from transcription, compared to the relative frequencies with which these Gs mutate to the other three bases, following the same color scheme as in (**a**). The number of cancers is equal to the number of mutable Gs. The thickness of each arrow is proportional to percent mutation frequencies, and the dual role of genotoxins is indicated. The data are derived from Tables 1-3.

Figure 2a is a visual description of the fate of G mutations in all cancers (including lung) compared to lung cancers (Tables 1, 2, 3), and Figure 2b depicts the "mutation flow" or relative frequencies with which mutations of G are generated during transcription and mutate to the other three bases, all of which result in cancer. The proposed dual effects of genotoxins on transcription and on base damage are also noted.

When mutations of C were analyzed (Table 3), relatively small differences were seen in the fate of total mutable Cs due to lung cancer C mutations. While C-to-T mutations predominate in the data sets of both lung and all types of cancers, proportionally fewer (7.9%) occur in the former case.

A similar analysis (Tables 4, 5, 6) compares *p53 *mutations in all cancers to colon cancers. In contrast to lung mutations, in which G-to-T mutations predominate, the most frequent G mutation in colon cancer is G-to-A. Also, while the relative number of G mutations increase to a minor extent in lung (4.6%), they decrease in colon cancer (2.0%). However, a significant increase (11.9%) occurs in the relative number of mutable Cs, suggesting an association with the development of colon cancer. Moreover, the *fate *of mutable Gs and Cs is in striking contrast to that seen in lung cancer mutations. The fate of mutable Cs is primarily to Ts, while there are relatively more G-to-A default mutations compared to G-to-T mutations. However, as all of these mutation fates are associated with cancer, they do not alter the frequency with which it occurs. The increased availability of background mutable Cs may be critical to the increased frequency of colon cancer. These results also suggest that a different mutagenic mechanism is operative in colon versus lung cancer.

**Table 4:** Mutations in colon compared to all cancers (including colon) in *p53 *codons 210–290

	A	**C**	**G**	T
Total number of all mutations in 240 nt (6662)	865	**1875**	**3375**	547
Percent of total	13.0%	**28.1%**	**50.7%**	8.2%
				
Number of colon mutations in 240 nt (651)	42	**260**	**317**	32
Percent of colon	6.4%	**40.0%**	**48.7%**	4.9%
				
Difference in total mutations compared to colon mutations	-6.6%	**+11.9%**	**-2.0%**	-3.3%

Analyzed as in Tables 1-3 for colon cancer.

**Table 4 **shows the total number of all mutations (including colon) compared to colon mutations and the propensity of each of the four bases to mutate to any other base (with mutations of both G and C in bold).

**Table 5:** Mutations of G to other bases in *p53 *codons 210–290

	**A**	C	**T**
Total G mutations including colon (3375)	**2082**	351	**942**
Percent of total	**61.7%**	10.4%	**27.9%**
			
Colon G mutations (317)	**257**	19	**41**
Percent of colon	**81.1%**	6.0%	**12.9%**
			
**Difference in total G mutations due to the presence of colon G mutations**	**+19.4%**	-4.4%	**-15.0%**

Analyzed as in Tables 1-3 for colon cancer.

**Table 5 **shows the ultimate fate of G mutations in all cancers due to colon cancers.

**Table 6:** Mutations of C to other bases in *p53 *codons 210–290

	A	G	**T**
Total C mutations including colon (1875)	157	187	**1531**
Percent of total	8.4%	10.0%	**81.6%**
			
Colon C mutations (260)	8	10	**242**
Percent of colon	3.1%	3.8%	**93.1%**
			
**Difference in total C mutations due to the presence of colon C mutations**	-5.3%	-6.2%	**+11.5%**

Analyzed as in Tables 1-3 for colon cancer.

**Table 6 **shows the ultimate fate of C mutations in all types of cancers due to the presence of colon cancers.

Results from the analyses of lung cancer mutations in *p53 *codons 210–290 (Tables 1, 2, 3) prompted a similar examination of 15 hypermutable bases in codons 213, 220, 245, 248, 249, 273, and 282 (the first two positions of all codons plus the third position of codon 249) (Tables 7, 8, 9). Note that the evolution of hypermutable bases has resulted in twice as many mutable G sites as C sites in this more selective hypermutable subset, and that there is a clear increase (10.7%) in the number of G mutations, again suggesting a causal relationship with the incidence of lung cancer. As in Table 2, compensatory shifts are seen in the percent of Gs that mutate to A versus T in lung cancer mutations compared to total mutations (Table 8). The data show a 34.5% increase in G-to-T and a 6.6% increase in G-to-C mutations in lung cancers, accompanied by a 41.1% decrease in G mutations to A. This is consistent with a shared source of mutable Gs, as seen in Table 2. Partitioning the fates of mutable Cs to the other bases also occurs (Table 9), but the effects are minor.

**Table 7:** Mutations in lung compared to all cancers in fifteen hypermutable bases of *p53*

	A	C	**G**	T
Total number of all mutations in 15 nt (3292)	225	1195	**1857**	15
Percent of total	6.8%	36.3%	**56.4%**	0.5%
				
Number of lung mutations in 15 nt (407)	26	107	**273**	1
Percent of lung	6.4%	26.3%	**67.1%**	0.2%
				
Difference in total mutations compared to lung mutations	-0.4%	-10.0%	**+10.7%**	-0.3%

These hypermutable bases consist of the first two positions of codons 213, 220, 245, 248, 273, and 282 and all positions in codon 249.

The composition of the bases analyzed is 2 As, 4 Cs, 8Gs, and 1T. **Table 7 **shows the total number of all mutations (including lung) compared to lung mutations and the propensity of these mutable bases to mutate to any other base (with mutations of G in bold).

**Table 8:** Mutations of G to other bases in fifteen hypermutable bases of *p53*

	**A**	C	**T**
Total G mutations including lung (1857)	**1214**	107	**536**
Percent of total	**65.3%**	5.8%	**28.9%**
			
Lung G mutations (273)	**66**	34	**173**
Percent of lung	**24.2%**	12.4%	**63.4%**
			
**Difference in total G mutations due to the presence of lung G mutations**	**-41.1%**	+6.6%	**+34.5%**

These hypermutable bases consist of the first two positions of codons 213, 220, 245, 248, 273, and 282 and all positions in codon 249.

The composition of the bases analyzed is 2 As, 4 Cs, 8Gs, and 1T. **Table 8 **shows the ultimate fate of G mutations in all cancers due to lung mutations (with G-to-A and G-to-T in bold).

**Table 9:** Mutations of C to other bases in fifteen hypermutable bases of *p53*

	A	G	T
Total C mutations including lung (1195)	17	49	1129
Percent of total	1.4%	4.1%	94.5%
			
Lung C mutations (107)	3	9	95
Percent of lung	2.8%	8.4%	88.8%
			
Difference in total C mutations due to the presence of lung C mutations	+1.4%	+4.3%	-5.7%

These hypermutable bases consist of the first two positions of codons 213, 220, 245, 248, 273, and 282 and all positions in codon 249.

The composition of the bases analyzed is 2 As, 4 Cs, 8Gs, and 1T. **Table 9 **shows the ultimate fate of C mutations in all cancers due to the presence of lung cancers.

In *p53*, an excellent correlation is seen between observed and predicted mutation frequency for the non-transcribed strand (Fig. 1), while no correlation exists for the transcribed strand. Thus, while a C-to-A transversion on the transcribed strand could occur to produce a T in the non-transcribed strand via mismatch repair, this mechanism probably makes a small contribution to the results of our analyses.

Codons 248 and 273 play a critical role in DNA binding, in addition to having the highest predicted and observed mutation frequencies in the *p53 *mutation database [17]. These codons were therefore chosen to further refine our analyses of the hypermutable bases. The results are seen in Tables 10, 11, 12, and a comparison with Tables 7, 8, 9 indicates that the characteristics of these two codons are typical of those seen for the other hypermutable lung cancer analyses.

**Table 10:** Mutations in lung compared to all cancers in hypermutable codons 248 and 273

	A	C	**G**	T
Total number of mutations in 4 nt (1775)	0	728	**1047**	0
Percent of total	0	41.0%	**59.0%**	0
				
Number of lung mutations in 4 nt (187)	0	63	**124**	0
Percent of lung	0	33.7%	**66.3%**	0
				
Difference in total mutations compared to lung mutations	0	-7.3%	**+7.3%**	0

These hypermutable bases consist of the first two positions of codons 248 and 273.

The composition of the bases analyzed is 2 Cs and 2Gs. **Table 10 **shows the total number of mutations (including lung) compared to lung mutations and the propensity of these mutable bases to mutate to any other base (with total mutations of G in bold).

**Table 11:** Mutations of G to other bases in hypermutable codons 248 and 273

	**A**	C	**T**
Total G mutations including lung (1047)	**863**	31	**153**
Percent of total	**82.4%**	3.0%	**14.6%**
			
Lung G mutations (124)	**46**	9	**69**
Percent of lung	**37.1%**	7.3%	**55.6%**
			
**Difference in total G mutations due to the presence of lung mutations**	**-45.3%**	+4.3%	**+41.0%**

These hypermutable bases consist of the first two positions of codons 248 and 273.

The composition of the bases analyzed is 2 Cs and 2Gs. **Table 11 **shows the ultimate fate of G mutations in all cancers due to lung mutations (with G-to-A and G-to-T in bold).

**Table 12:** Mutations of C to other bases in hypermutable codons 248 and 273

	A	G	T
Total C mutations including lung (728)	11	21	696
Percent of total	1.5%	2.9%	95.6%
			
Lung mutations (63)	3	2	58
Percent of lung	4.8%	3.2%	92.1%
			
Difference in total C mutations due to the presence of lung C mutations	+3.3%	+0.3%	-3.5%

These hypermutable bases consist of the first two positions of codons 248 and 273.

The composition of the bases analyzed is 2 Cs and 2Gs. **Table 12 **shows the ultimate fate of C mutations in all cancers due to the presence of lung cancers.

## Conclusion

These studies indicate that genotoxin-induced G-to-T base damage to *p53 *is not correlated with the incidence of lung cancer, which must therefore be due to a different mechanism. We propose that mild base damage by genotoxins provokes the stress response during which *p53 *promoter elements are activated, leading to transcription, hypermutation, the loss of p53 regulatory functions, and ultimately to cancer. A variety of experimental evidence supports this mechanism: (a) genotoxins induce upregulation of *p53 *transcription [9,21-24]; (b) persistently upregulated *p53 *expression is associated with the accumulation of *p53 *mutations and tumorigenesis [19,20]; (c) intrinsically mutable unpaired bases in secondary structures are formed during transcription [26,29,30]; and (d) an excellent correlation exists (Fig. 1) between observed mutation frequencies in *p53 *and base mutability predicted by a model of transcription-induced mutagenesis [17]. Although these experimental lines of evidence do not address the possibility of a dual role for carcinogens in mutagenesis, the insights obtained from Tables 1, 2, 3, 4, 5, 6, 7, 8, 9, 10, 11, 12 provide strong support for the existence of two different, as well as independent mechanisms induced by genotoxins. The newly discovered relationships described above suggest that the same mechanism regulates the number of mutable bases in lung cancer as in all cancers, and that this mechanism (transcription) differs from that regulating the *fate *of the mutable bases, e.g., the number of available mutable Gs that mutate to T versus the number that mutate to A. Further, the data describe the two independent roles of genotoxins: first, they upregulate *p53 *transcription and increase the availability of mutable bases, which determines the incidence of cancer, and second, they inflict base damage, e.g., increase the ratio of G-to-T versus G-to-A mutations, which has no affect on the frequency of cancer. These analyses are consistent with the conclusion that transcription-exposed mutable bases determine the incidence of cancer, which is also the conclusion of an independent analysis of hypermutable bases using the computer algorithm, *mfg *(Fig. 1). The development of cancer clearly involves a complex series of mutagenic and selective events. It now appears that these events may include the participation of transcription-driven secondary structures and intrinsically mutable bases encoded within the *p53 *gene.

## Competing interests

The author(s) declare that they have no competing interests.
